# Supplementary material for: Mutations in PCYT2 disrupt etherlipid biosynthesis and cause a complex hereditary spastic paraplegia
Source: Brain. 2019 Oct 22;142(11):3382–97. doi: 10.1093/brain/awz291 (PMC6821184; doi:10.1093/brain/awz291)
Supplement: awz291_Supplementary_Data [file awz291_supplementary_data.zip › awz291-Suppl_data/Supplementary_Data4.zip!/Supplementary information 3 - fibroblast lipidomics.zip!/Fibroblast lipidomics/Controle vs Patient1/Statistical plots and data/PC species/Volcano plot_alternative.pdf]

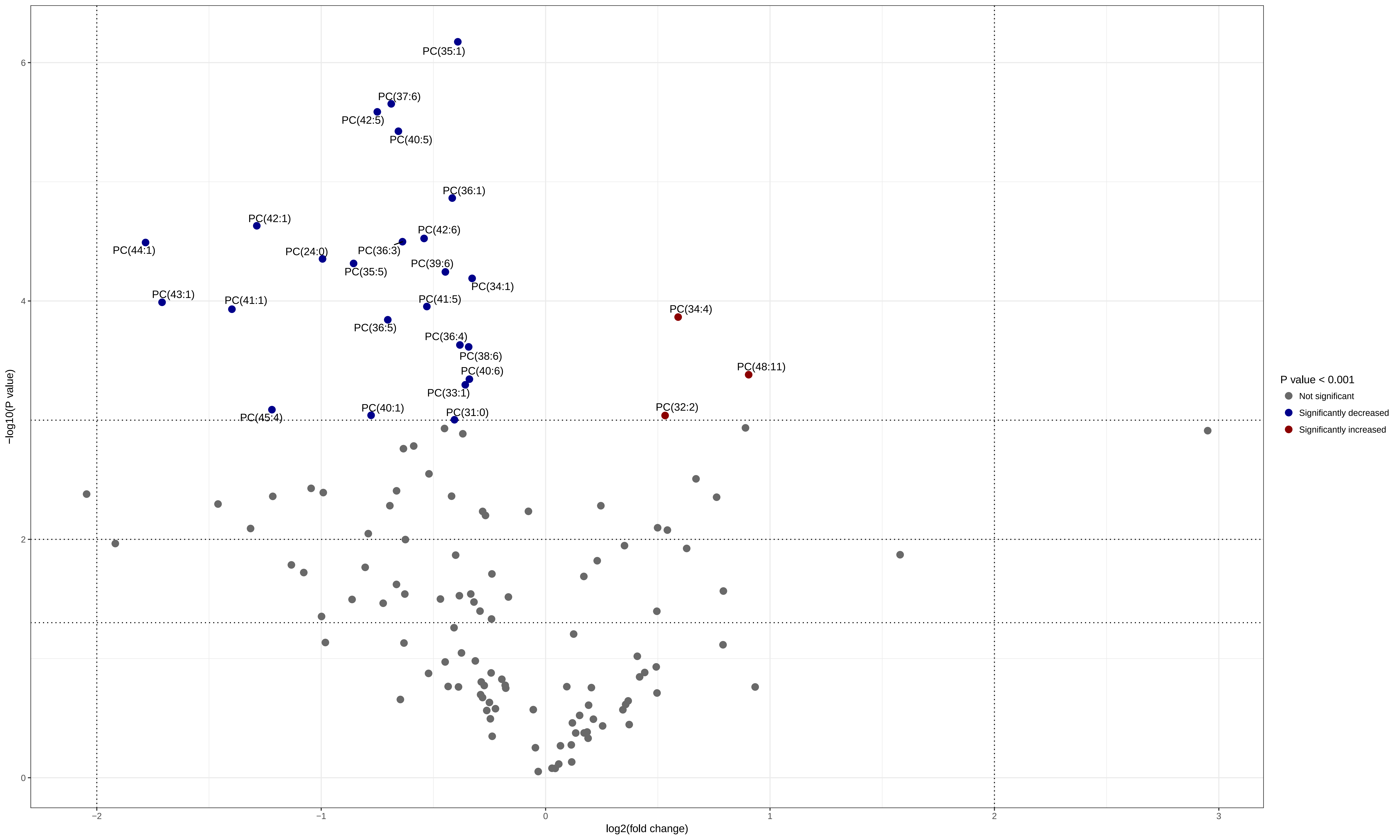

PC(35:1)
PC(37:6)
PC(42:5)
PC(40:5)
PC(36:1)
PC(42:1)
PC(42:6)
PC(44:1)
PC(24:0)
PC(36:3)
PC(39:6)
PC(35:5)
PC(34:1)
PC(43:1)
PC(41:1)
PC(41:5)
PC(34:4)
PC(36:5)
PC(36:4)
PC(38:6)
PC(48:11)
PC(40:6)
PC(33:1)
PC(40:1)
PC(31:0)
PC(32:2)
PC(45:4)
−log10(P value)
log2(fold change)
P value < 0.001
Not significant
Significantly decreased
Significantly increased
